# Case of Cancer

**Published:** 1843-08

**Authors:** A. H. Buchanan

**Affiliations:** Nashville, Tennessee


### WESTERN JOURNAL

OF

### MEDICINE AND SURGERY.

AUGUST, 1843.

ART. I.—*Case of Cancer.*—Reported to the Medical Society of Tennessee, May, 1843. By A. H. BUCHANAN, M.D., of Nashville, Tennessee.

Thomas Terrell (of Columbia, Tennessee), aged forty-nine, a man of more than ordinary intelligence, of good form, and apparently of sound constitution, gives the following history of his case.

"When I was a boy between twelve and thirteen years of age, I discovered a small tumor about the size and feel of a large squirrel shot, situated under the skin at the root of the nose, just where it joins the forehead and mid-way between the eyes. The tumor remained stationary, without any pain, until I was about twenty years of age. In the course of the next five years it increased to the size of a common rifle ball without pain, and in 1821, it was extirpated by Dr. McConnell, of Lancaster, Kentucky. The wound immediately healed, but the tumor soon returned and increased with greater rapidity than it had done at any previous time, and on the 23d day of December, 1823, it had attained to the size of a small almond, and burst open the cicatrice of the old wound. The tumor was now removed by Dr. Smith, President of New-Haven College, and resembled in appearance a large black-berry. The wound again healed in ten or twelve days; but the tumour returned in a short time and increased until the fall of 1824, when it was again removed by Dr. Kincade, of Shelbyville, Tennessee. The tumor, when operated on by Dr. Kincade, was rather less than before the previous operation, but was situated nearer to the internal angle of the left eye. The incisions again healed, but in the spring of 1825, the same surgeon found it necessary to repeat the operation, and removed the tumour which had returned, together with some hard callous growth in its neighborhood. During all this time the tumour excited no pain. The wound as heretofore healed readily, but the tumour returned and gradually increased until the winter of 1826, when I was seized with a violent attack of influenza, so that my life was despaired of by my physicians. But I recovered gradually from this attack, and found that it had a salutary effect upon the tumour, which almost entirely disappeared for seven or eight months, during which time my health was quite delicate. In 1827, I moved to St. Charles in Missouri, and the tumour again commenced increasing rapidly. Dr. Stodder was now consulted and he pronounced the disease to be cancerous, and advised me not to submit to any further operations. He applied *potash of red-oak bark, and drew out the plug with a plaster of tar*, which arrested the progress of the tumour, and the part assumed nearly a healthy appearance for about five years.

In 1831 I was married; I have one child, now about eight years of age, who has no signs of the disease.

In 1832 my disease again commenced increasing, and several small hard lumps or knots appeared under the old cicatrice. In the spring of 1833 it was burnt with *wet fire* and never afterwards healed, but gradually spread and increased in size; the ulcerated parts were frequently filled with calomel which had rather a bad effect. In the fall of 1839 Professor Dudley, of Lexington, was consulted; he gave me no satisfactory advice, but declined operating, and stated that operations would injure me. I returned home and the tumour has gradually increased to this date, November 1841; as you see, it is now the size of a child's head of a year old, and hollowed out in its centre; about two years ago the tumour was not larger than a small apple."

Having taken down with my pencil the above history, given by Mr. Terrell himself, I now examined the tumour, which was one of the most frightful I ever beheld; he really looked as if he had two heads, and the weight of the tumour was so great that when he sat up, it drew the head forwards. The centre of the tumour was excavated and was very deep, so that the bones of the nose were entirely destroyed; and also the external lamina of the os frontis had ulcerated through at several points, the pits being large enough to receive the point of the finger. All the surface of the cavity, was covered with a very offensive secretion, more resembling a yellowish cream than any thing else. The surface was also very irregular and knotty, and when this secretion was removed by washing, or fell off spontaneously, which it frequently did, the surface looked perfectly clean and of a pinkish color, with several pits upon it, and with numerous blood-vessels, both arteries and veins, creeping over it; some of the arteries could be seen distinctly pulsating. In a few days after the removal of the above secretion, it was again renewed, and remained firmly attached for ten or twelve days, or longer, when it would again come off, deepening and widening at each time the internal cavity. This process of ulceration opened one or more blood vessels for the first time in the month of May, 1841, from which a profuse hemorrhage occurred; and from that time at different intervals of two or three weeks or longer, hemorrhages occurred of greater or less severity.

The external surface of the tumour was very irregular and knotty, though smooth and shining. The tumour was also firm and elastic, and had upon its surface numerous large and tortuous veins creeping over it, which were filled with a very dark blood, while the blood in the arteries, which were also numerous, was very florid, so that they were readily distinguished from the veins. But the shining surface of the tumour through which these vessels were so distinctly traced would sometimes become incrusted with a dark colored scurf, which would remain for a few days and drop off, leaving the surface as bright as before. The tumour, or rather tumours, occupied each side of the nose, and were in fact the superior eyelids enormously distended and infiltrated, being very firm and hard, and each one as large at least as the fist, extending down over the eye-balls so as completely to obstruct the vision. These tumours met above on the os frontis and there projected forwards so that there were three raw edges, one above, and one on each side of the nose; these edges turned out, and, in dressing the cavity, frequently bled profusely. Sometimes the bleeding could be anticipated with a great deal of certainty from the progressive ulceration of the raw edge towards the blood-vessels in its neighborhood. Terrell also mentioned that he could tell with much certainty when a hemorrhage was about to occur, as the tumour always felt heavy and full and somewhat painful previous to an attack, and that invariably he was troubled with an erection of the virile member and an emission of semen, ten or twenty hours before the rupture of a blood-vessel. I also noticed a very evident difference at times in the fulness of the blood-vessels upon the tumour, sometimes they were enormously distended and at others quite flaccid. In the course of the fall Terrell had at times suffered severely from pain in the eyes, produced most probably by the pressure of the distended lids; he also complained of a weight and uneasiness in the front part of the head. But at no time did he complain of the peculiar lancinating and darting pains which are said to be evidences of cancer. His general health was not much impaired, and he indulged in the use of a moderate amount of food, though his skin was excessively pale and almost transparent from the frequent loss of blood. Such then was the condition of Terrell when I made the above notes, and when I thought he would not live many weeks, but he lingered on for twelve months longer, suffering greatly at times from the loss of blood, either from the internal surface of the tumour or from its raw edges. The cavity of the tumour was generally filled with cotton which was also stuck over the raw edges, and when removed, which was done every day, it was filled with the creamy matter above alluded to, and was horribly offensive. The edges of the tumour always bled more or less at each dressing, being perfectly raw and everted for half an inch or more on the lateral margins of the tumour, while the edge above turned under towards the cavity. Sometimes after a hemorrhage of some severity pearly looking drops were suspended from the most dependent edges of the tumour.

In the course of the ensuing summer of 1842, I was requested to see him in consequence of numerous maggots that were found in the ulcerated cavity; they were very large and in spite of the stimulating injections used to displace them, they revelled for several days in the deep fissures of the cavity, in the frontal sinuses, and in the spongy bones of the nose. During their stay the creamy matter accumulated in great quantities, and the stench emitted by it was intolerable. In the course of ten or twelve days they all or nearly all came away with the matter, which left the cavity perfectly clean and shining, and of a light pink color. The depth and size of the pits could now be seen very distinctly, and also numerous small arteries were visible, and could be noticed pulsating with ordinary force and regularity; one artery was as large as a crow-quill and appeared perfectly denuded. At this time while the cavity was clean there was but little odor from the tumour. Thus he continued, sometimes better and sometimes worse, throughout the summer and fall, but always looked as if he was upon the verge of the grave, and as if it was impossible to survive another attack of hemorrhage, yet they returned even to fainting, and still he would revive, while his bloodless surface looked almost transparent. Towards the close of the summer he became dropsical, and in the month of December, 1842, he expired.

Previously to his death he gave me permission to make a post-mortem examination of his body, and to preserve so much of his head as would be necessary to illustrate the disease.

Six hours after his death, in presence of most of the physicians of Columbia, I proceeded to make an examination. We first opened the chest and found the tissues within remarkably white and bloodless; there were slight adhesions between the pleura costalis and pulmonalis by ligamentous bands of old formation, but there were no signs of recent inflammation, and the lungs were remarkably sound; not a tubercle nor other evidence of disease was met with in their substance. The heart was rather smaller than common and was firmly contracted; the cavity of the pericardium was obliterated by a firm adhesion to the surface of the heart; the large blood-vessels issuing from the heart were all sound, except the aorta, which had upon its internal surface a deposit of osseous matter, about the size of the finger nail, just beyond the orifice of the right coronary artery; around the orifice of the left coronary artery there was also the commencement of osseous transformation. Around the ostium venosum, between the left auricle and ventricle at the fixed margin of the mitral valve, there was also a ring of osseous matter about two lines wide; the lining membrane of the aorta for an inch or more beyond the semilunar valves, was elevated into little pimples which appeared to be the commencement of ossific degeneration. The lymphatic glands under the sternum were healthy, but the black bronchial glands were much indurated. The mucous membrane of the trachea and bronchial tubes was sound. In our manipulations with the knife in the chest several veins were opened, from which flowed a very thin and florid blood which formed a firm coagulum; the medical gentlemen present, were requested to notice this fact as one worthy of record in a medico-legal point of view. We now opened the cavity of the abdomen, and, as in the chest, found the tissues very pale and bloodless; the peritoneum appeared healthy, and no adhesions existed between the surfaces of the intestines; the cavity of the peritoneum contained several pints of serum. The colon was very much contracted, not being more than an inch in diameter, the small intestines appeared of a natural size. The stomach was enormously distended and contained about four pints of water mixed with buttermilk; its internal surface was pale and had firmly attached to it a thick ropy mucus; the internal surface of the small intestines was free from disease. The liver was rather smaller, firmer and of a paler color than natural; the spleen of ordinary size and soft. The kidneys and bladder appeared to be sound. The contents of the cranium were subsequently examined: the cerebrum, cerebellum, and medulla oblongata, were sound in all their parts, as also their surrounding membranes, except that portion of the dura-mater opposite to the perforations in the os frontis, which at these points was converted into scirrhous tumors some of which projected through the openings in the bone. The anterior lobes of cerebum immediately behind these lumps were altered in their structure, and seemed to be absorbed from the pressure of two indurated lumps which projected in upon them from the internal surface of the dura-mater; these lumps were about the size of a pigeon's egg and attached to the substance of the anterior lobes. The anterior part of the ethmoid bone, the nasal bones, and the nasal processes of the superior maxillary bones, were all destroyed. The eye balls were hid beneath the anterior inferior surface of the projecting tumours, but by raising up these tumours the sclerotica in both eyes appeared to be sound, as did also the iris. I have brought to the society the preparation of the head and heart, which they can examine for themselves.

I will not detain the society by an examination of the question, whether cancer is a disease of constitutional or of local origin, although it is one of the utmost importance to be decided. From all I have seen written on this subject, however, I am disposed to adopt the opinion that it is a disease which in a large majority of instances, particularly in its early stages, may be effectually and permanently removed by the knife.

